# Using Adaptive Surrogate Models to Accelerate Multi-Objective Design Optimization of MEMS

**DOI:** 10.3390/mi16070753

**Published:** 2025-06-26

**Authors:** Ali Nazari, Armin Aghajani, Phiona Buhr, Byoungyoul Park, Yunli Wang, Cyrus Shafai

**Affiliations:** 1 Department of Electrical and Computer Engineering, University of Manitoba, Winnipeg, MB R3T 5V6, Canada; nazaria@myumanitoba.ca (A.N.); aghajana@myumanitoba.ca (A.A.); buhrdphb@myumanitoba.ca (P.B.); 2 Nanotechnology Research Centre, National Research Council Canada, Edmonton, AB T6G 2M9, Canada; byoungyoul.park@nrc-cnrc.gc.ca; 3 Digital Technologies Research Centre, National Research Council Canada, Ottawa, ON K1A 0R6, Canada; yunli.wang@nrc-cnrc.gc.ca

**Keywords:** MEMS, design optimization, surrogate modeling, Lorentz force actuator, thermal actuator, multi-objective optimization, online learning, Gaussian process regression, finite element method

## Abstract

This study presents a comprehensive multi-objective optimization framework specifically designed for micro-electromechanical systems (MEMS). The framework integrates both traditional and adaptive optimization techniques, named Surrogate-Assisted Multi-Objective Optimization (SAMOO) and Adaptive-SAMOO (A-SAMOO), respectively. By addressing key limitations of traditional approaches, such as the consideration of objective constraints and the provision of multiple design options, the proposed framework enhances both flexibility and practical applicability. Results show that adaptive optimization outperforms traditional offline methods by delivering a greater number and higher quality of optimal solutions while requiring fewer finite element method simulations. The adaptive approach showed a significant advantage by attaining high-quality solutions while requiring only 2.8% of the finite element method (FEM) evaluations compared to traditional methods that do not incorporate surrogate models. This performance boost highlights the advantages of online learning in enhancing the accuracy, speed, and diversity of solutions in MEMS optimization. These optimization schemes were tested on multiple MEMS devices with varying physics and complexities, specifically the U-shaped Lorentz force actuator, serpentine Lorentz force actuator, and thermal actuator. The results highlight the robustness and versatility of the proposed methods, particularly in addressing cases involving discrete design variables and strict objective constraints. This comprehensive, step-by-step framework serves as a valuable resource for researchers and practitioners aiming to optimize MEMS designs from the ground up, providing a reliable and effective approach to multi-objective optimization in MEMS applications.

## 1. Introduction

Micro-electromechanical systems (MEMS) have become indispensable in various devices such as biomedical apparatus [1,2], wearables [3], lasers [4], autonomous vehicles [5], drones [6], smartphones [7], and telescopes [8,9]. Their compact size, energy efficiency, high sensitivity, and ease of integration and control make them highly attractive for diverse applications [10]. To maximize MEMS performance, design parameter (e.g., geometric structure, material properties, and actuation mechanisms) optimization is generally conducted before fabrication. Evolutionary algorithms (EAs) can be used to explore the design space, with finite element method (FEM) analyses employed to derive objective values [11,12]. Many optimization algorithms demand a large number of evaluations [13,14]. However, FEM evaluations can become computationally intensive, particularly when multiple physical aspects are involved; even if a single MEMS simulation takes only a few minutes [15], evaluating thousands of designs could take days or even weeks. For such challenges, it is essential to employ an efficient optimization strategy that can find optimal solutions using only a limited number of expensive evaluations.

One of the primary approaches to mitigate this issue is to replace the computationally expensive function with a more efficient one that approximates the original problem with acceptable accuracy. This substitute is referred to as a “surrogate model” or “meta model” in the literature. An optimization procedure that employs this method is known as “surrogate-assisted optimization (SAO)” or “surrogate-based optimization (SBO)” [16,17]. Surrogate models are simplified mathematical approximations that replicate the behavior of the actual system based on a limited number of high-fidelity FEM simulations. A single full-scale FEM simulation of a MEMS may take minutes to complete, depending on mesh density and physical complexity. In contrast, once a surrogate model is trained using a representative dataset (e.g., 50–100 FEM samples), it can evaluate new design points in milliseconds. This enables rapid prediction of performance metrics (objectives)—such as displacement, resonant frequency, or stress—without the need to repeatedly run expensive simulations. As a result, surrogate models drastically reduce the total optimization time from days to minutes, making them particularly effective for iterative design processes and large-scale parametric sweeps.

Despite significant advancements in the application of SBO to MEMS design optimization [18,19,20], several shortcomings remain to be addressed. First, many studies focus on single-objective optimization, which does not address the complexity of integrating multiple design objectives [21,22,23]. Second, traditional SAO methods often utilize static surrogate models—constructed from a fixed set of data—which may fail to provide sufficient accuracy in regions of the design (exploration) space that yield optimal or near-optimal solutions. This level of inaccuracy can significantly degrade the precision and reliability of optimization outcomes. Third, many MEMS SAO frameworks fail to adequately manage objective constraints imposed by specific application requirements [24,25]. Lastly, despite using surrogates, some approaches still face high computational costs due to the need for extensive initial simulations to build accurate models.

In our previous study [26], surrogate-based optimization was applied to a U-shaped Lorentz force actuator, focusing on the impact of preprocessing techniques and sampling size on the number of optimal designs identified. Building on this work, the present study introduces an adaptive optimization scheme and evaluates its effectiveness through the design optimization of three distinct MEMS devices.

This study employs Surrogate-Assisted Multi-Objective Optimization, leveraging a limited number of simulations to train surrogate models using datasets of varying sizes and preprocessing techniques. An evolutionary algorithm is utilized to identify optimal design configurations, ensuring that the optimization objectives adhere to predefined constraints. To enhance the process further, an adaptive approach is integrated, continuously refining the surrogate models for greater accuracy. These methods are designated as Surrogate-Assisted Multi-Objective Optimization (SAMOO) and Adaptive Surrogate-Assisted Multi-Objective Optimization (A-SAMOO), respectively.

The primary contribution of this study lies in the development and validation of a generalized multi-objective optimization method suitable for diverse MEMS applications. Specifically, our contributions include the following:

1. Adaptive Optimization: We incorporate adaptive optimization, dynamically updating surrogate models throughout the process. This ensures that the models remain accurate as new data is integrated, overcoming limitations associated with static surrogate models.
2. Multi-Objective Optimization: Unlike many existing studies that focus on single-objective optimization, our approach considers multiple design objectives, capturing the complexity and trade-offs inherent in MEMS design. At the end of the optimization, the user is provided with a set of non-dominated solutions representing the optimal trade-offs between conflicting objectives, allowing users to explore and select the design that best aligns with their specific requirements or preferences.
3. Constraint Handling: Our framework takes into account constraint handling to ensure that the optimization process adheres to the specific requirements and limitations of each MEMS application.
4. Validation Across Multiple Devices: The proposed optimization methods have been systematically applied to three distinct MEMS actuators—namely, the U-shaped Lorentz force actuator, the serpentine Lorentz force actuator, and the thermal actuator. This comprehensive validation ensures the robustness and versatility of the approach, highlighting its potential applicability across a wide range of MEMS designs.

The paper structure is as follows: Section 2 details the SAMOO algorithm, followed by Section 3, that describes the A-SAMOO technique. Section 4 discusses the operational principles and optimization results for various actuators. Lastly, Section 5 summarizes the key findings of this work.

## 2. Surrogate-Assisted Multi-Objective Optimization (SAMOO)

The following steps are taken in the SAMOO algorithm:

1. Initial sampling of the design space: A relatively small number of designs are sampled and evaluated using computationally expensive simulations to obtain their respective objective values.
2. Preprocessing: Objectives are scaled using various methods to study the effect of preprocessing on the quality of surrogates.
3. Surrogate modeling: Surrogate models are trained on the scaled datasets to imitate the original model. This model now “predicts” the performance of each design. Based on the prediction accuracy of surrogates trained on differently scaled data, the best model is chosen to advance to the next step.
4. Optimization: An optimization algorithm is utilized to obtain the optimal solutions using the trained model as the evaluation function. The optimal solutions found are evaluated via FEM to ensure their actual performance is close enough to their predicted value calculated by the surrogate.

Since the surrogate evaluations are much faster to compute, the optimizer can use a large number of function evaluations without consuming heavy compute and time resources. Each step of this process is detailed in the subsequent sections.

### 2.1. Initial Sampling of the Design Space

To construct an accurate surrogate model, it is crucial to generate a set of training samples that uniformly cover the entire design space to ensure proper representation of the actual model; while random sampling is computationally inexpensive, it often fails to ensure proper coverage. A more effective approach is Latin hypercube sampling (LHS) [27] and it works as follows. Suppose that we are to sample *n* points from the design space. First, each dimension of the design space is split into *n* sections. LHS chooses the samples so that if we were to exit the design space from each dimension along the hypercubes, we would not encounter another sample [28]. However, LHS alone does not guarantee a space-filling property. To address this, the maximin criterion [29] can be applied alongside LHS. This criterion seeks to maximize the minimum distance between points, promoting an even distribution across the design space.

While larger sampling sizes improve model accuracy, they also increase computational costs. Thus, the choice of sample size requires balancing model accuracy with computational efficiency. In this work, we employ LHS with the maximin criterion to generate a representative sample set. Simulations are performed using COMSOL Multiphysics version 6.2 on a Mac Studio equipped with an M1 Ultra processor and 64 GB of RAM.

### 2.2. Preprocessing

The preprocessing of objectives for the training of surrogate models, as well as its role in the optimization process, is a relatively unexplored area in the surrogate-assisted optimization of MEMS. In this study, three different preprocessing techniques were considered for the objectives before integrating them into the surrogate models:

- Normalization, defined as
  (1) $$y_{norm}=\frac{y-y_{min}}{y_{max}-y_{min}}$$
  This equation scales the objectives between 0 and 1, using the minimum and maximum values from the initial samples. However, because optimization results may extend beyond this initial range, some objectives might end up with negative scaled values. This can complicate the evaluation of surrogate modeling and optimization outcomes. To address this, 1 was added to the scaled objectives to ensure all values remain positive, resulting in ranges between 1 and 2.
- Log transformation where values undergo natural logarithm transformation. For instance, if the value of the resonant frequency objective is 4000 Hz, the scaled value would be 8.29.
- Log transformation followed by normalization.

### 2.3. Surrogate Modeling

In our study, we used Gaussian process regression (GPR) as the surrogate model. GPR assumes that the data originates from a Gaussian process, which consists of a set of random variables with a joint Gaussian distribution [30]. This assumption allows GPR to model complex, non-linear relationships between inputs and outputs effectively. Unlike traditional regression methods that provide only point estimates, GPR provides estimation of the uncertainty of its predictions, typically represented as variance or standard deviation. This capability is particularly valuable in scenarios where understanding the confidence in predictions is as important as the predictions themselves.

GPR models a function $f\left(x\right),x\in R^{d}$ based on noisy observations y=f(x)+ϵ, with f(x) and noise ϵ following Gaussian distributions $N(0,k\left(x,x^{\prime }\right))$ and $N(0,\sigma_{\epsilon }^{2})$, respectively. The correlation between values at x and $x^{\prime }$ is defined by the covariance function (kernel). Here, we use the squared exponential kernel:

(2) $$k\left(x,x^{\prime }\right)=\sigma_{f}^{2}\;exp(-\frac{1}{2\lambda^{2}}|x-x^{\prime }{|}^{2})$$

where $\sigma_{f}^{2}$ is the variance and λ is the length-scale hyperparameter. Given *n* training observations $\left\{X_{t},y_{t}\right\}$, where $X_{t}\in R^{n\times d}$ and $y_{t}\in R^{n}$, the predictions for new inputs $X_{★}\in R^{m\times d}$ have mean and variance

(3) $$mean\left(f_{★}\right)=K_{★,t}{\left[K_{t}+\sigma_{\epsilon }^{2}I\right]}^{-1}y_{t}$$

(4) $$var\left(f_{★}\right)=K_{★}-K_{★,t}{\left[K_{t}+\sigma_{\epsilon }^{2}I\right]}^{-1}K_{t,★}$$

in which $K_{★,t}\in R^{m\times n}$ is the covariance matrix between each pair of new and training inputs, $K_{t}\in R^{n\times n}$ is the covariance matrix between each pair of training inputs, and $K_{t,★}\in R^{n\times m}$ is the covariance matrix between each pair of training and new inputs. Each element of covariance matrices, denoted as K(i,j), represents the covariance between the i-th and j-th points calculated via Equation (2). $I\in R^{n\times n}$ is also the identity matrix. In practice, Equation (3) is used as the prediction. The hyperparameters λ, $\sigma_{f}^{2}$, and $\sigma_{\epsilon }^{2}$ (denoted as θ) are optimized by maximizing the log marginal likelihood:

(5) $$log\;p\left(y|X,\theta \right)=-\frac{1}{2}y^{T}K_{y}^{-1}y-\frac{1}{2}log\left|K_{y}\right|-\frac{n}{2}\;log\left(2\pi \right)$$

where $K_{y}=K_{t}+\sigma_{\epsilon }^{2}I$ [31].

In this study, we implement GPR using the Scikit-learn (v1.6.1) Python framework [32], training a separate surrogate model for each objective. To evaluate the predictive accuracy of the surrogate models, we use the mean absolute percentage error (MAPE), defined as

(6) $$MAPE=\frac{1}{n}\sum_{i=1}^{n}\left|\frac{y_{t}-y_{p}}{y_{t}}\right|,$$

where $y_{t}$ and $y_{p}$ refer to the true and predicted values of an objective, respectively. MAPE is chosen because it expresses the average error as a percentage, making it easy to compare errors across objectives with different scales. After calculating MAPE for each model, the one with the lowest prediction error is selected for the subsequent optimization stage.

### 2.4. Optimization

A multi-objective optimization (MOO) procedure can be defined as [33]

(7) $$\begin{array}{cccc} \; & \mathrm{Minimize}\;f_{m}\left(x\right), & m & =1,2,\ldots ,M;\\ \; & \;\mathrm{subject}\;\mathrm{to}:\\ \; & \;\;g_{j}\left(x\right)\geq 0, & j & =1,2,\ldots ,J;\\ \; & \;\;h_{k}\left(x\right)=0, & k & =1,2,\ldots ,K;\\ \; & \;\;x_{i}^{\left(L\right)}\leq x_{i}\leq x_{i}^{\left(U\right)}, & i & =1,2,\ldots ,n. \end{array}$$

in which *M*, *J*, *K*, and *n* represent the number of objectives, inequality constraints, equality constraints, and design variables, respectively. A solution *x* in the design space, $R^{n}$, is considered “feasible” if it satisfies both the inequality and equality constraints and falls within the specified bounds. For each solution *x*, there exists a corresponding point $y=f\left(x\right)\in R^{M}$ in the objective space.

Evolutionary algorithms (EAs) have been widely used for MEMS design optimization [11,34,35,36]. The appeal of EAs lies in their versatility in solving MOO problems due to their ability to handle diverse variable types (continuous, combinatorial, or mixed) without assumptions on constraints, convexity or differentiability of the objectives [37,38].

We use Non-dominated Sorting Genetic Algorithm II (NSGA-II) [39], a popular evolutionary algorithm, for optimization. NSGA-II ranks solutions using non-dominated sorting and selects parents for crossover and mutation based on crowding distance, maintaining diversity in solutions [40]. The algorithm iteratively improves solutions over generations, balancing diversity and convergence.

In traditional MOO, each function evaluation requires an FEM analysis, leading to high computational costs. However, in SAMOO, the surrogate model would be utilized to calculate objective values with great computational efficiency. NSGA-II implemented in the Pymoo Python library [41] is used in our work.

Quality indicators (QIs) are essential for assessing the results of multi-objective optimization. A key measure of solution quality is the ability to approximate the Pareto front, which consists of all non-dominated solutions. A solution is Pareto-optimal if no other solution can improve one objective without worsening another [42]. The hypervolume (HV) indicator is a common QI, representing the n-dimensional volume between a solution set and a reference point:

(8) $$HV\left(A\right)=\lambda \left(\underset{a\in A}{⋃}\left\{x|a≺x≺r\right\}\right),$$

where λ is the Lebesgue measure. There are multiple reasonable selections for the reference point such as the nadir and worst objective points (vectors with three or more objectives). When multiple solution sets are to be compared with respect to each other, a common practice is to combine these sets and chose the reference point from this constructed set [43]. We use the worst objective point of the combined solution set as the reference point for hypervolume calculation.

Following the optimization process, we obtain a set of non-dominated solutions. However, since these designs have been evaluated using surrogate models, validating them through actual simulations is essential to determine their objective values accurately. We report the number of these non-dominated solutions that meet the predefined objective constraints. Additionally, to assess the accuracy of our optimization, we measure the discrepancy between the surrogate-evaluated solutions and their true objective values using MAPE.

## 3. Adaptive Surrogate-Assisted Multi-Objective Optimization (A-SAMOO)

In SAMOO, a static surrogate model was built using an initial set of samples. This approach has a drawback: optimal solutions often lie in specific, narrow regions of the design space. Consequently, many samples might end up in areas that do not make meaningful contribution to the optimization procedure; wasting computational resources. Through online learning, A-SAMOO aims to mitigate this issue by focusing efforts on the most promising regions, reducing computational costs and improving surrogate model accuracy in regions it is most needed. The primary distinction of A-SAMOO from SAMOO lies in its dynamic sampling strategy, utilized by the NSGA-II optimizer outlined in Section 2.4.

**Figure 1:**
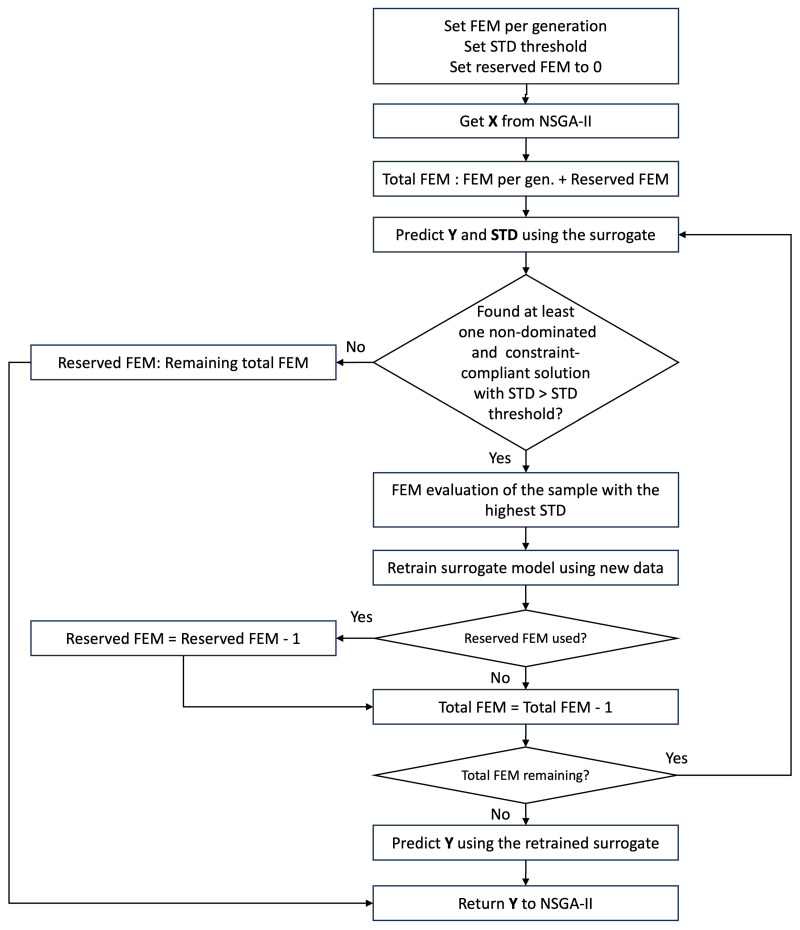
Flowchart of A-SAMOO integration with NSGA-II.

Figure 1 shows the mechanism of A-SAMOO. Within the NSGA-II optimization loop, the GPR surrogate model estimates the objectives and their uncertainties, expressed as standard deviations. Among the Pareto-optimal solutions that satisfy the constraints, the solution with the highest uncertainty is selected for evaluation assuming that its standard deviation is larger than a threshold. The combination of Pareto-optimality, constraint compliance, and large standard deviation ensures sampling of only promising regions.

The threshold serves two primary purposes. First, it ensures that FEM evaluations are only conducted when the surrogate model’s uncertainty is high, preventing evaluation of samples that would add minor new information to the optimization process. Second, due to the configuration of the GPR—especially the chosen kernel—training samples typically have very low standard deviations, indicating high confidence in the predictions. The threshold prevents these designs from being re-evaluated, as their uncertainties fall far below the threshold.

Once a solution undergoes FEM evaluation, the resulting objectives are used to update and refine the surrogate model. When multiple evaluations are planned within a generation, each sample is evaluated one at a time, and after each FEM simulation, the surrogate model is updated accordingly. This sequential method is deliberate and particularly effective in early generations when model uncertainties are higher, as each evaluation can significantly adjust the predictions; while this approach does increase the frequency of surrogate model training and prediction steps, these operations are computationally inexpensive compared to FEM simulations. Thus, the additional computational overhead is minimal or even negligible. After all evaluations are completed, the updated predictions are used to guide NSGA-II in ranking and selection of individuals that proceed to the next generation.

## 4. Results

SAMOO and A-SAMOO methodologies were evaluated on three different MEMS devices: the U-shaped Lorentz force actuator, serpentine Lorentz force actuator, and the thermal actuator. Our investigation focused on these devices alone and did not consider additional elements, such as the mechanics of the substrate, since we are wishing to develop the methodology for particular actuator designs. LHS is employed to generate five distinct datasets within the design space, each comprising 20, 50, 100, 200, and 400 samples. Five-fold cross-validation is used to assess the prediction accuracy of the surrogate models. The parameters associated with NSGA-II optimization algorithm are listed in Table 1. For A-SAMOO, the number of FEM evaluations per generation is set to 1. Furthermore, when objectives are scaled using a combination log scaling and normalization, the standard deviation threshold is 1 × 10^−4^, found via a trial-and-error process.

### 4.1. U-Shaped Lorentz Force Actuator

**Figure 2:**
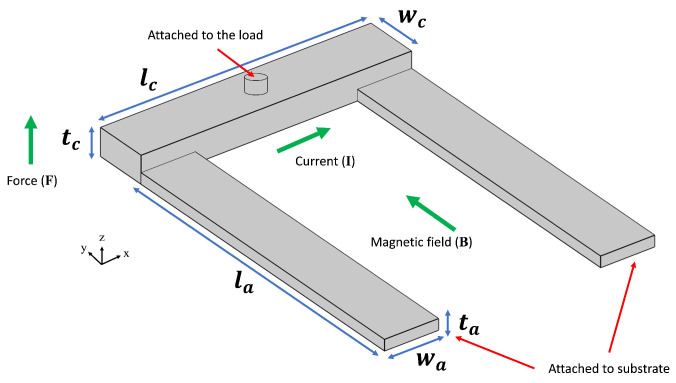
Schematic of the Lorentz force actuator.

The first MEMS device considered for optimization is a U-shaped Lorentz force actuator [44], as depicted in Figure 2. When exposed to an external magnetic field, a current passing through the crossbar generates a Lorentz force, causing the crossbar, and hence the load, to move vertically. Mathematically, the generated force is calculated using the equation

(9) $$\left|\vec{F}\right|=\left|I\vec{L}\times \vec{B}\right|=BIL\cdot \sin\theta$$

where $\vec{F}$ is the generated Lorentz force, *I* is the current, $\vec{L}$ is the length of the crossbar ($l_{c}$ in Figure 2), $\vec{B}$ is the magnetic field, and θ is the angle between the magnetic field and the crossbar. In this work, the magnetic field is assumed to be provided via a permanent magnet in a way that the provided magnetic field is perpendicular to the crossbar (sin 90° = 1).

Since these actuators have applications for adaptive optics, the load is considered to be a mirror membrane composed of Au/Si_3_N_4_/Au layers. Based on the volume and density of these layers, the mirror mass is computed. The mirror is then modeled as a mass and spring system attached to the crossbar via a small pillar depicted in Figure 2. The opposite ends of the two cantilever springs are mechanically clamped to the substrate. A thin-film Al layer, covering the entire structure, provides the pathway for the current. The actuator itself is made of polycrystalline silicon. The magnetic field (B) required for the operation is assumed to be provided externally. This value is used for the calculation of required current and voltage. The vertical displacement of the crossbar in one direction is set to a fixed value, and hence, the total displacement of the cross bar is twice the value.

The meshing size for the simulations was carefully chosen to balance convergence and computational efficiency. Larger mesh sizes were found to occasionally fail due to significant inaccuracies in the results, making them unsuitable for reliable optimization. On the other hand, smaller mesh sizes did not lead to noticeable changes in results but significantly increased computation times. More specifically, the chosen meshing sizes are tested to be small enough that finer sizes do not make significant changes to the simulation results. This is due the fact that the main contribution of this work is the optimization process itself and time-consuming sampling would only hinder extensive studies.

A trial-and-error process was performed to obtain the meshing configuration that balanced the convergence and consistency of the simulations and the computational times. Meshing is calibrated for “General physics” and set to “Predefined Coarse” size. The average simulation time was 6.02 s. The parameters used in the modeling and their values are listed in Table 2.

The design is defined by seven geometric design variables, each with specific ranges outlined in Table 3. The optimization process focuses on three conflicting objectives: minimizing von Mises stress, minimizing reaction force, and maximizing resonant frequency [45]. The structure undergoes a solid mechanics analysis to determine the resulting stress and the required reaction force. This approach enables the calculation of both the force necessary to achieve the specified deformation and the stress experienced by the structure. Resonant frequency is simulated through eigenfrequency study. Constraints applied to these objectives are detailed in Table 4.

**Figure 3:**
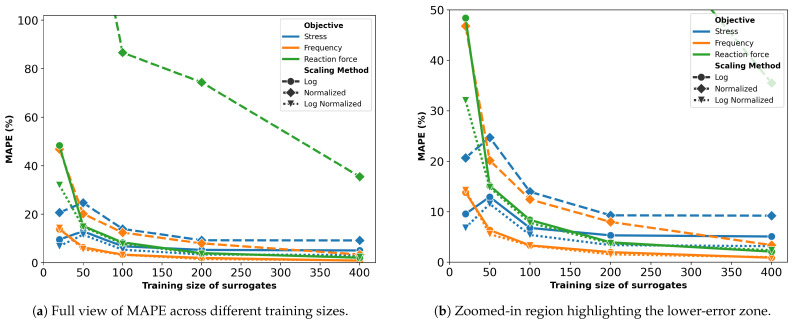
MAPE of surrogate models for the U-shaped actuator with varying training sizes and preprocessing. (**a**) Shows the full plot; (**b**) highlights the low-MAPE region.

Figure 3 displays the prediction performance of the surrogates, trained on varying sample sizes, in terms of MAPE. To improve readability, the y-axis is capped at 100%. As expected, the error decreases with larger training sizes, as they provide more information for the model.

Surrogates trained on normalized objectives perform the worst, especially for the reaction force, with a best MAPE of 35.5%. For the other two objectives, errors are lower but still higher compared to other scaling methods. Surrogates trained using log scaling or a combination of log scaling with normalization yield a similar performance, with a slight advantage in favor of the latter. Thus, only these surrogates are used in the optimization process.

**Figure 4:**
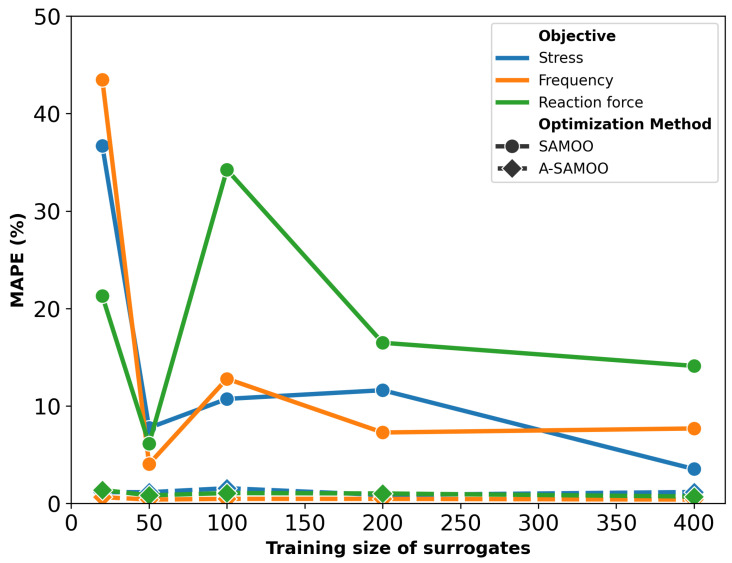
MAPE after optimization for the U-shaped Lorentz force actuator.

After optimization is conducted using both SAMOO and A-SAMOO, the resulting solutions are evaluated through simulation to assess their quality. Figure 4 demonstrates the MAPE of all objectives after the optimization for both algorithms. As it can be viewed, the error for A-SAMOO is significantly lower. For the sampling size of 200, for instance, the MAPE values are just 0.87%, 0.47%, and 1.02% for stress, frequency, and reaction force, respectively. This can be explained by the fact that optimal solutions or likely concentrated in a small region of the design space and the static modeling technique simply leaves those regions under-sampled. However, A-SAMOO, updates the model in promising regions that could benefit from higher accuracy. Another important outcome is that the optimizer is less vulnerable to being misled by inaccurate surrogate predictions to sub-optimal directions. It is noteworthy that A-SAMOO, even with the smallest surrogate model (size 20), still performed significantly better in terms of accuracy compared to the largest model used in SAMOO, which was trained on 20 times more data points.

**Figure 5:**
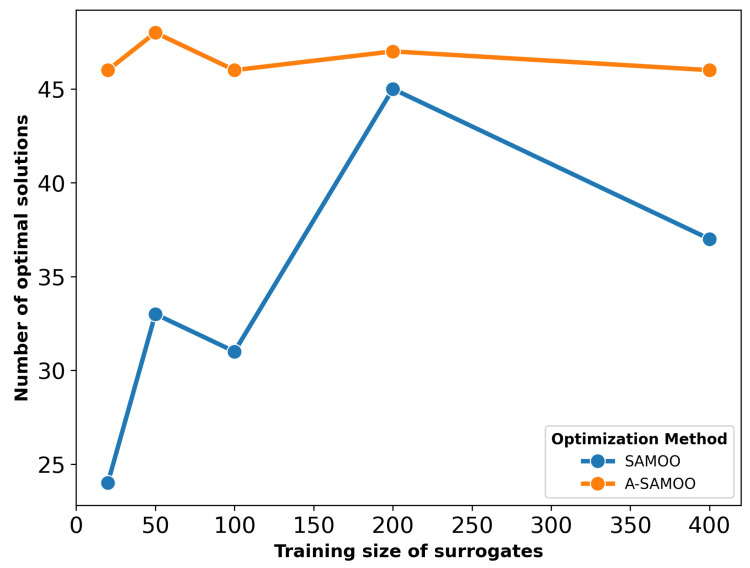
Number of true optimal solutions for the U-shaped Lorentz force actuator.

The number of optimal solutions produced is another critical aspect of optimization results. Both SAMOO and its adaptive variant predicted that all the solutions they returned are optimal. The term optimal refers to the union of solutions that are within objective constraints and are Pareto-optimal (non-dominated with respect to each other). However, Figure 5, which depicts the number of optimal solutions, suggests otherwise.

Once again, A-SAMOO performs significantly better, as the number of its optimal solutions is very close to the maximum possible, the population size of 50. In contrast, the numbers are lower for SAMOO. This discrepancy is due to the lower prediction quality of its surrogate model.

**Figure 6:**
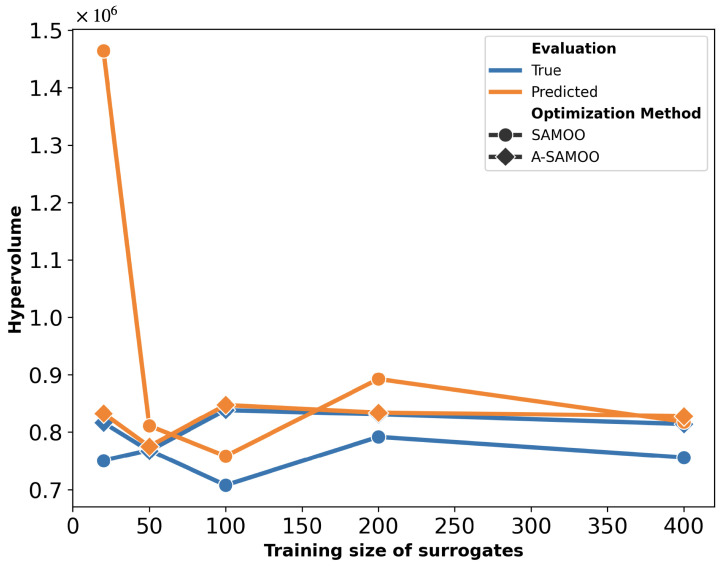
Hypervolume of predicted solutions for the U-shaped Lorentz force actuator.

The hypervolume quality metrics for the solution sets of both algorithms are shown in Figure 6. The “Predicted” values represent the hypervolume measured immediately after optimization, while the “True” values correspond to the results after FEM re-evaluation. This plot can be interpreted from two perspectives. Firstly, for each scheme, the predicted hypervolume exceeds its true value, indicating that the optimizer overestimated solution quality. This discrepancy is notably larger for SAMOO, especially with smaller training sizes. In contrast, A-SAMOO’s predicted and true hypervolumes nearly overlap, suggesting that online learning significantly aids the optimizer in providing a more accurate estimate of its results. Secondly, regarding the hypervolume values themselves, those for A-SAMOO are consistently higher, indicating that the quality of its solutions is superior. Similar to the observations in Figure 5, even the smallest adaptive model outperforms the largest surrogate model used in the other algorithm.

### 4.2. Serpentine Lorentz Force Actuator

**Figure 7:**
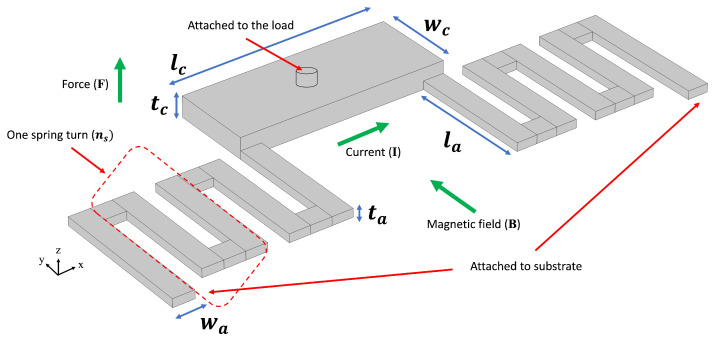
Schematic of the serpentine Lorentz force actuator.

The second MEMS studied in this work is a more complex version of the actuator in Section 4.1 with the distinction that the crossbar is attached to serpentine structures that act as springs. Hence, the MEMS device is called the serpentine Lorentz force actuator, illustrated in Figure 7. This actuator has an additional discrete design variable $n_{s}$ corresponding to a number of spring turns that ranges from 1 to 10. Three new objectives, operational temperature difference compared to ambient condition (25 °C or 298 K), current, and voltage participate in the optimization, all of which to be minimized. Eigenfrequency and solid mechanics studies are similar to those of the U-shaped actuator mentioned in Section 4.1. The thermal solution is only considering Joule heating and thermal conduction since it is a DC current flow and very small temperature differential. Due to the additional complexity and the introduction of thermal study, the average simulation time is longer compared to that of the U-shaped actuator, being 26.2 s. The objectives and their allowed ranges are listed in Table 5.

**Figure 8:**
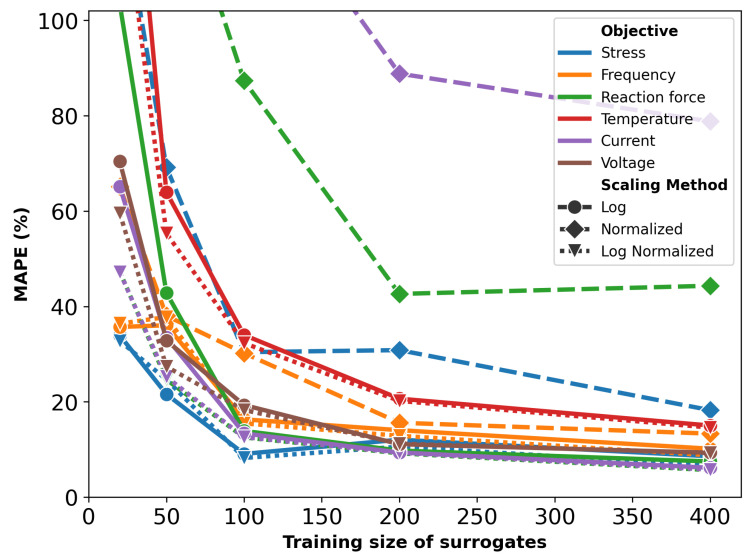
MAPE of surrogate models with different training sizes and preprocessing for the serpentine Lorentz force actuator. To improve readability, the y-axis is capped at 100%.

The mean absolute percentage error (MAPE) for surrogates trained on various sampling sizes and preprocessing methods are plotted in Figure 8. Similar to the U-shaped actuator, the combination of log scaling and normalization produced the most accurate models.

However, there are key differences compared to Figure 3. First, the actuator with serpentine springs shows generally higher error values due to its increased complexity, which demands more data for accurate modeling. Second, unlike the simpler U-shaped actuator, resonant frequency is not the most accurately predicted objective for the more complex Lorentz force actuator. The addition of serpentine springs has increased the complexity of frequency modeling more than that of stress and reaction force.

**Figure 9:**
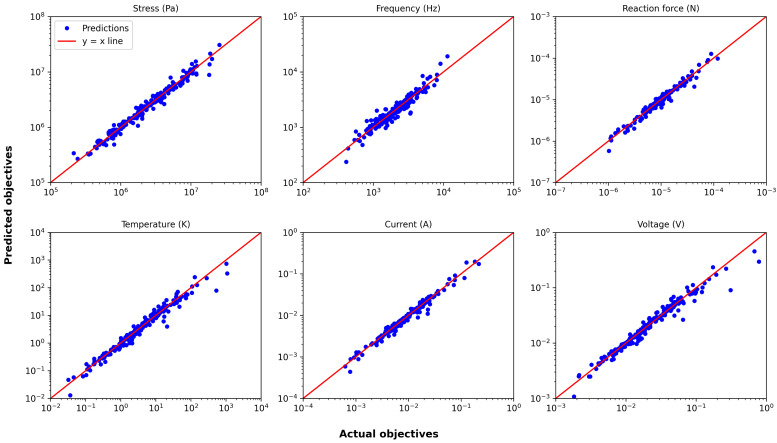
Actual vs. predicted plot for the serpentine Lorentz force actuator with a training size of 200.

Figure 9 presents, as an example, the actual versus predicted plot for the surrogate model trained with the best-performing scaling method and a sample size of 200. Most data points lie close to the y=x line, indicating reasonable predictive accuracy despite the increased complexity.

**Figure 10:**
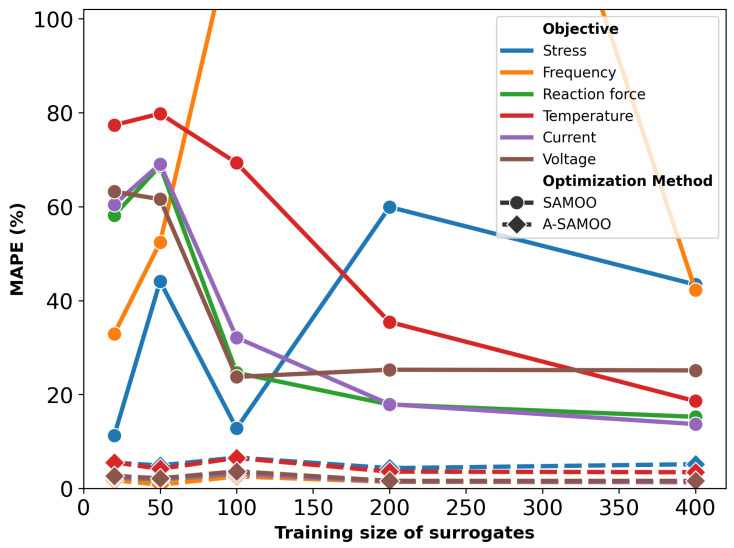
MAPE after optimization for the serpentine Lorentz force actuator. To improve readability, the y-axis is capped at 100%.

As shown in Figure 10, the error values for SAMOO solution sets after optimization are excessively high. This results from two main factors: the use of initial lower-quality surrogates compared to those for the U-shaped actuator, and the additional design variable and complexity. A-SAMOO generates much more accurate results, though the errors remain higher than those in Figure 4.

**Figure 11:**
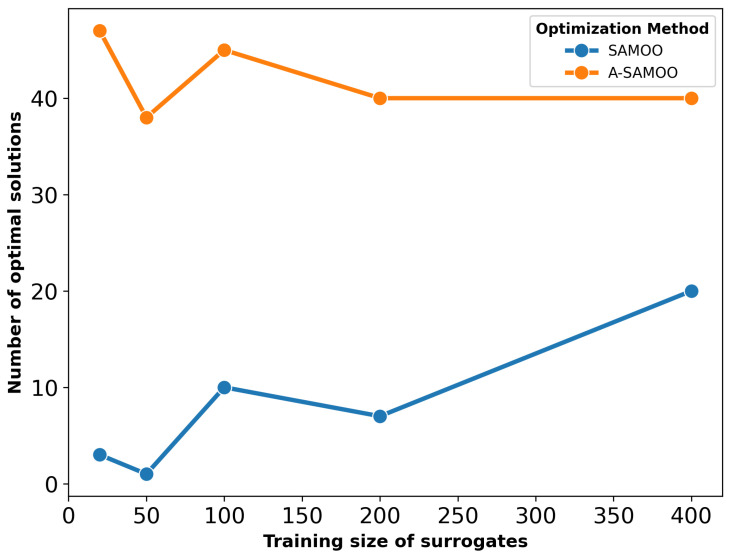
Number of true optimal solutions for the serpentine Lorentz force actuator.

**Figure 12:**
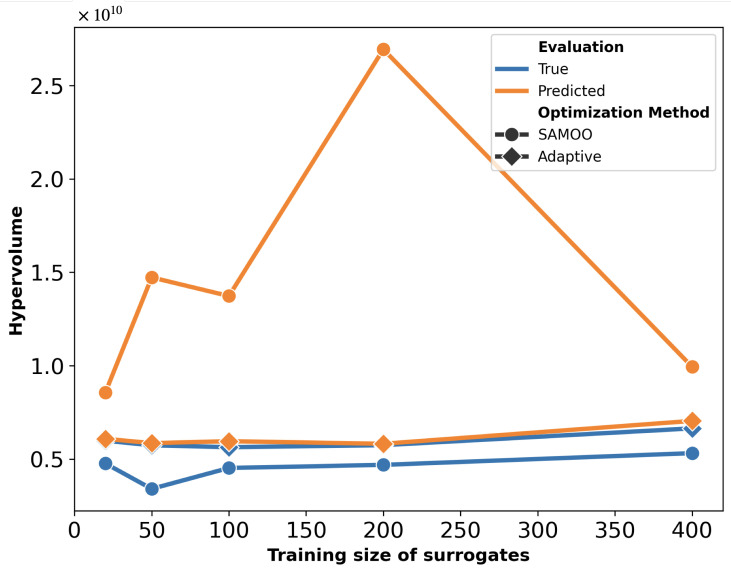
Hypervolume of predicted solutions for the serpentine Lorentz force actuator.

The substantial improvement in optimization results achieved by A-SAMOO over SAMOO is further supported by the higher number of desirable solutions and the greater hypervolume, as shown in Figure 11 and Figure 12, respectively.

### 4.3. Thermal Actuator

**Figure 13:**
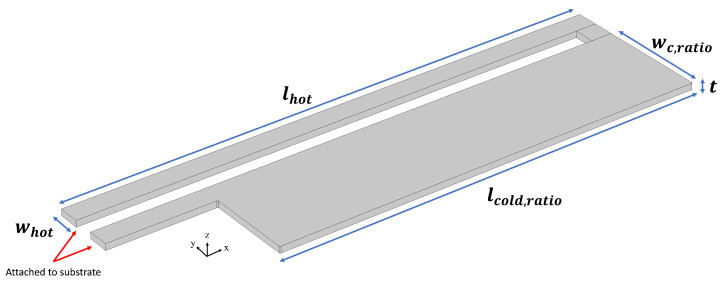
Schematic of the thermal actuator.

Another MEMS considered for the optimization in this work is the thermal actuator. As it can be seen in Figure 13, the actuator consists of two primary hot and cold arms. The difference in cross-sectional area of the arms mean that the narrower one reaches significantly higher temperatures and as a result, expands to a larger degree. This differential expansion results in a bending motion, and hence, deformation, which is the core functionality of such devices.

Similar to Lorentz force actuators, the material chosen for the thermal actuator structure is polycrystalline silicon, with the same parameters as those listed in Table 2. The width and length of the junction between the two arms is equal to the width of the hot arm.

At micro-scale, heat transfer via radiation is negligible [46,47]. Furthermore, conduction dominates convection [48]. Hence, for the thermal modeling, it is assumed that the only form of heat transfer is via conduction to the substrate. Meshing type is set to “Physics-controlled” with the automatic size of “Extra fine”. The average computation time was 22 s.

Six design variables, provided in Table 6, are considered. The width and length of the cold arm are determined via ratios of those of the hot arm. By using ratios, we can easily adjust the relative dimensions of the cold arm to modify the structure geometries without being constrained by absolute measurements. This also allows us to assess our optimization scheme in cases where some design variables are ratios and not geometric variables.

Five objectives are chosen for the optimization, shown in Table 7. Temperature refers to the temperature difference of the hottest spot in the structure, under operation, to that of the ambient condition. Similar to Lorentz force actuators, all objectives are to be minimized, except for the resonant frequency and deformation.

**Figure 14:**
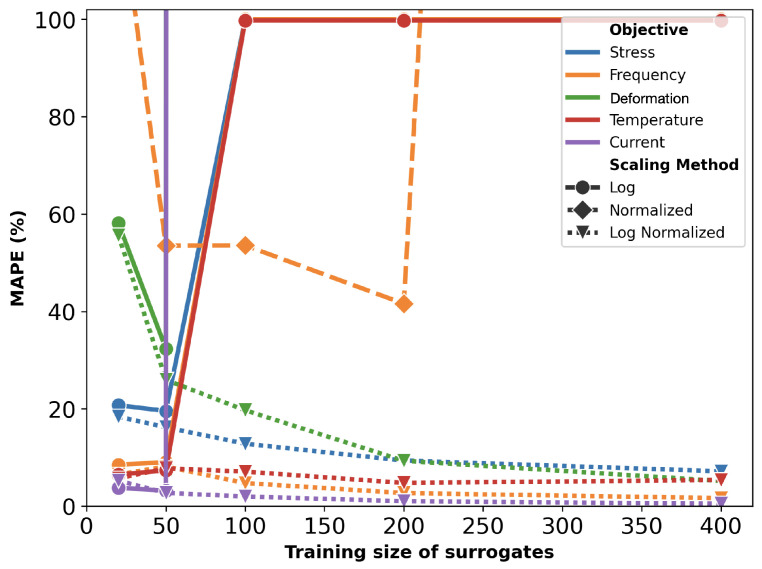
MAPE of surrogate models with different training sizes and preprocessing for the thermal actuator. To improve readability, the y-axis is capped at 100%.

Figure 14 illustrates the MAPE for surrogate models trained with varying dataset sizes for the thermal actuator. The choice of scaling method significantly influences the predictive accuracy of the surrogate models. Error values corresponding to log scaling and normalization are generally larger than 100% and therefore do not fit in the plot. Similar to previous cases, the combination of log scaling and normalization consistently provides the most accurate predictions.

**Figure 15:**
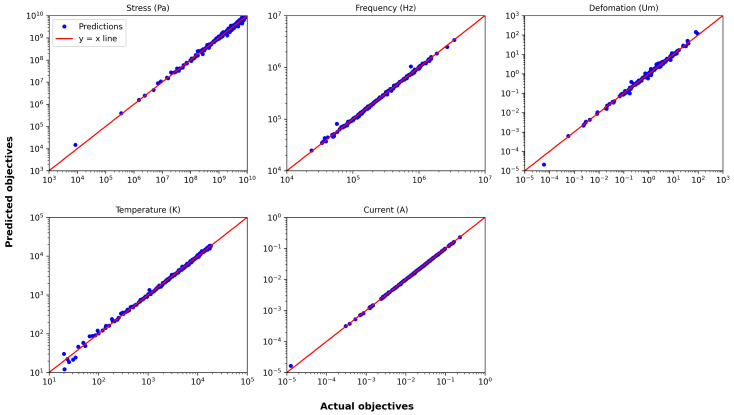
Actual vs. predicted plot for the thermal actuator with a training size of 200.

In contrast to previous actuators, the thermal actuator does not benefit as much from log scaling alone. This difference is likely attributable to the considerably broader range of objectives associated with the thermal actuator, where most objectives span at least three orders of magnitude, excluding the resonant frequency; while the specific reasons for the heightened impact of normalization remain unclear, it is evident that the wide range of values plays a critical role in shaping the predictive performance. As an example, actual vs. predicted plots of objectives for surrogates with the training size of 200 are provided in Figure 15, demonstrating accurate predictions, particularly for current.

**Figure 16:**
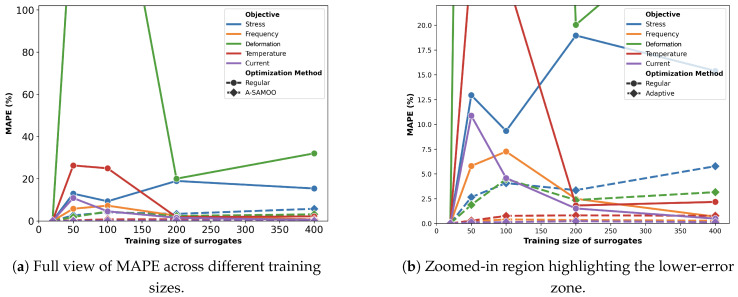
MAPE after optimization for the thermal actuator. The y-axis is capped at 100% for clarity. (**a**) shows the complete plot, while (**b**) provides a zoomed-in view of the low-error region.

The MAPE values after optimization are provided in Figure 16. With a training size of 20 samples, the optimizer fails to produce any optimal designs, making MAPE reporting irrelevant—an issue not previously encountered, even with adaptive optimization. This can be attributed strict objective constraints. For instance, the average stress value of initial samples was 2.82 × 10^9^ Pa, nearly 10 times that of the constraint. For deformation, the average was 5.29 μm, only slightly better than the constraint, 5 μm.

**Figure 17:**
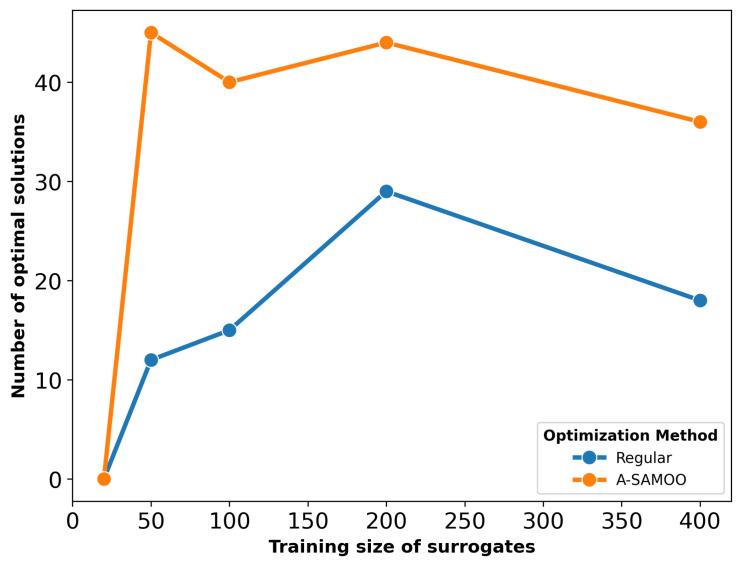
Number of true optimal solutions for the thermal actuator.

Figure 17 shows the number of optimal solutions versus training sizes for surrogates used in optimization. The results reaffirm that online training in the A-SAMOO approach enables the NSGA-II optimizer to find more optimal solutions compared to offline training.

**Figure 18:**
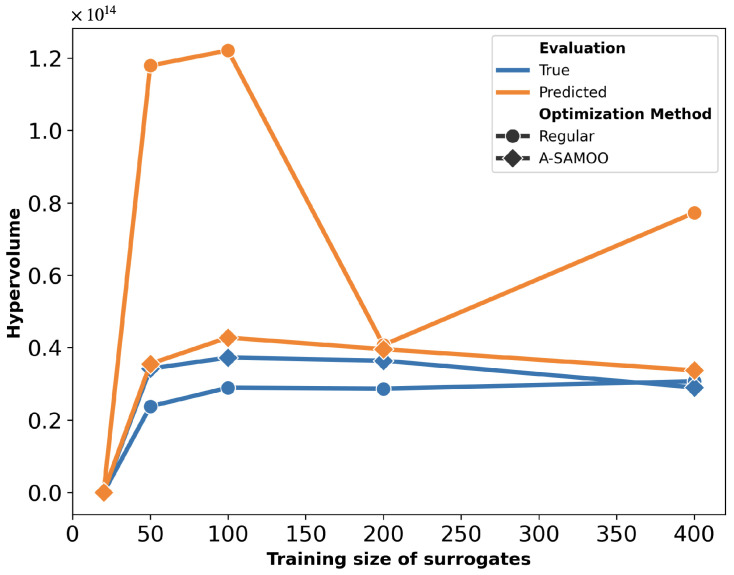
Hypervolume of predicted solutions for the thermal actuator.

Figure 18 further supports the advantages of A-SAMOO through hypervolume analysis. Most training sizes show a large discrepancy between actual and predicted hypervolume values for SAMOO, suggesting that offline-trained surrogates tend to overestimate solution quality. An anomaly occurs at a training size of 400, where the actual hypervolume for regular optimization slightly surpasses that of the adaptive approach. This difference is minor and does not undermine A-SAMOO’s overall benefits. It likely arises from inherent surrogate inaccuracies, which can occasionally lead to favorable outcomes.

Surrogate inaccuracies generally lower actual hypervolumes. However, in this instance, the inaccurate prediction (e.g., predicting 180 MPa stress instead of 200 MPa) aligned favorably with the optimization objectives. This pattern, where some predicted values outperform actual results, is consistently observed across all actuators, as seen in the actual versus predicted plots.

As summarized in Table 8, our work advances the state of the art in surrogate-assisted MEMS optimization by addressing several key limitations of previous studies; while most existing methods rely on static surrogate models, single-objective formulations, or lack proper constraint handling, our framework introduces an adaptive, multi-objective optimization approach (A-SAMOO) with integrated constraint management. By continuously refining the surrogate model during optimization, we ensure improved accuracy in the most critical regions of the design space. Furthermore, we validate our method across three distinct MEMS actuators, highlighting its robustness and general applicability. This comprehensive strategy not only enhances optimization reliability but also provides designers with a diverse set of Pareto-optimal solutions tailored to application-specific requirements.

## 5. Conclusions

This study addresses some of the gaps in the MEMS surrogate-assisted optimization literature by providing a robust, comprehensive, and step-by-step multi-objective optimization framework tailored specifically for these applications. It guides researchers seeking to accelerate their design process from scratch. Unlike most previous studies that relied on static, offline surrogate models, our work integrates adaptive optimization methods, including both regular (SAMOO) and adaptive (A-SAMOO) approaches. The adaptive method, in particular, demonstrated a clear advantage by consistently outperforming the static approach in terms of the quality and quantity of optimal solutions, requiring as low as 2.8% (70 vs. 2500) of FEM evaluations compared to traditional approaches that do not utilize surrogate models. This performance boost highlights the potential of online learning to enhance accuracy, speed, and the diversity of solutions in MEMS optimization.

The framework was rigorously tested across three distinct actuators, each presenting unique challenges in terms of design variables, objectives, and complexity. This validation across diverse devices illustrates the versatility and robustness of our approach. Notably, the framework successfully managed the stringent objective constraints of the thermal actuator, where many initial designs failed to meet stress and deformation limits.

Additionally, the proposed framework provides multiple optimal design options for MEMS designers, contrasting with many studies where multi-objective results are simplified into a single averaged solution. This feature offers greater flexibility and choice in the design process. Our findings also underscore the importance of careful preprocessing of objectives. It was demonstrated that appropriate choice of preprocessing ensured more accurate surrogate model predictions and hence, superior optimization results.

## Figures and Tables

**Table 1:** NSGA-II optimization parameters.

Parameter	Value
Population size	50
Offspring size	50
Number of generations	50
Crossover Probability	0.9
Crossover eta	20
Mutation eta	20

**Table 2:** Parameters used for modeling of Lorentz force actuators in COMSOL Multiphysics.

Parameter	Value	Unit
Crossbar vertical displacement in one direction	1	μm
Al layer thickness	1	μm
External magnetic field	0.7	T
Mirror area	1 × 1	mm
Mirror Au layer thickness (each one)	0.15	μm
Mirror Si_3_N_4_ layer thickness	0.5	μm
Au density	19,300	kg/m^3^
Si_3_N_4_ density	2850	kg/m^3^
Calculated mirror mass	7.215 × 10^−9^	kg
**Polycrystalline silicon parameters**		
Heat capacity at constant pressure	678	J/(kg·K)
Density	2320	kg/m^3^
Thermal conductivity	34	W/(m·K)
Young’s modulus	160 × 10^9^	Pa
Poisson’s ratio	0.22	-

**Table 3:** Design variables of the U-shaped Lorentz force actuator.

Variable	Symbol	Lower Bound	Upper Bound	Unit
Arm length	$l_{a}$	200	500	μm
Arm width	$w_{a}$	20	200	μm
Arm thickness	$t_{a}$	5	20	μm
Crossbar length	$l_{c}$	500	3000	μm
Crossbar width	$w_{c}$	50	400	μm
Crossbar thickness	$t_{c}$	25	50	μm
Load spring constant	$k_{m}$	1	10	Nm^−1^

**Table 4:** Optimization objectives of the U-shaped Lorentz force actuator.

Objective	Constraint	Unit
Stress	N/A	Pa
Resonant frequency	Higher than 5 × 10^3^	Hz
Reaction force	Lower than 2 × 10^−5^	N

**Table 5:** Optimization objectives of the serpentine Lorentz force actuator.

Objective	Constraint	Unit
Stress	N/A	Pa
Resonant frequency	Higher than 5 × 10^3^	Hz
Reaction force	Lower than 2 × 10^−5^	N
Temperature difference	Lower than 10	K
Current	N/A	A
Voltage	N/A	V

**Table 6:** Design variables of the thermal actuator.

Variable	Symbol	Lower Bound	Upper Bound	Unit
Hot arm length	$l_{hot}$	100	500	μm
Cold/hot arm length ratio	$l_{cold,ratio}$	0.2	0.9	-
Hot arm width	$w_{hot}$	3	30	μm
Cold/hot width ratio	$w_{cold,ratio}$	1.1	10	-
Thickness	*t*	1	5	μm
Voltage	*v*	0.01	10	V

**Table 7:** Optimization objectives of the thermal actuator.

Objective	Constraint	Unit
Stress	Lower than 3 × 10^8^	Pa
Resonant frequency	N/A	Hz
Deformation	Higher than 5	μm
Temperature difference	Lower than 800	K
Current	N/A	*A*

**Table 8:** Comparison of this work with existing literature.

Article	# Features	# Objectives	Surrogate	Optimizer	Notes
[49]	3	2	CNN	NSGA-II	Electrostatic micromotor; image inputs to surrogate
[18]	4	2	Kriging	NSGA-II	Comb-drive; cross-validation of surrogate
[21]	4	1	Kriging	DE	Calorimetric wall shear stress sensor; results verified with a redundant optimizer
[19]	9	1	Kriging	DE	Shell-type dome actuator, corrugated membrane actuator; promising regions highly populated
[23]	7	1	Kriging	Customized DE	Corrugated membrane actuator; SQP-based sampling, constraint handling with penalty factor
[22]	4, 10	1, 1	Kriging	DE	Thermal actuator, corrugated membrane actuator; weighted constraint violation sum as objective
[25]	5	5	Kriging	Desirability function approach, Gradient descent	Capacitive accelerometer; multi-objective, constraint handling, sensitivity analysis; no user-defined choice
[20]	4	3	Regression tree	NSGA-II	RF-MEMS suspended inductor, RF-MEMS switch; multi-objective optimization with multiple Pareto-optimal outputs
**This work**	**6, 7, 8**	**3, 5, 6**	**Gaussian process regression (Kriging)**	**NSGA-II**	**U-shaped Lorentz force actuator, actuator with spring arms, thermal actuator; multi-objective, adaptive optimization, Pareto-optimal set, constraint handling, and preprocessing study**

## Data Availability

The original contributions presented in this study are included in the article. Further inquiries can be directed to the corresponding author.

## Abbreviations

The following abbreviations are used in this manuscript:

MEMS	Micro-electromechanical systems (MEMS)
SAMOO	Surrogate-Assisted Multi-Objective Optimization
A-SAMOO	Adaptive Surrogate-Assisted Multi-Objective Optimization
EA	Evolutionary algorithm
FEM	Finite element method
SAO	Surrogate-assisted optimization
SBO	Surrogate-based optimization
LHS	Latin hypercube sampling
GPR	Gaussian process regression
MAPE	Mean absolute percentage error
MOO	Multi-objective optimization
NSGA-II	Non-dominated Sorting Genetic Algorithm II
QI	Quality indicator
HV	Hypervolume
